# Coffee Consumption and All-Cause, Cardiovascular, and Cancer Mortality in an Adult Mediterranean Population

**DOI:** 10.3390/nu13041241

**Published:** 2021-04-09

**Authors:** Laura Torres-Collado, Laura María Compañ-Gabucio, Sandra González-Palacios, Leyre Notario-Barandiaran, Alejandro Oncina-Cánovas, Jesús Vioque, Manuela García-de la Hera

**Affiliations:** 1 Instituto de Investigación Sanitaria y Biomédica de Alicante, ISABIAL-UMH, 03010 Alicante, Spain; l.torres@umh.es (L.T.-C.); lcompan@umh.es (L.M.C.-G.); sandra.gonzalezp@umh.es (S.G.-P.); lnotario@umh.es (L.N.-B.); aoncina@umh.es (A.O.-C.); manoli@umh.es (M.G.-d.l.H.); 2 Unidad de Epidemiología de la Nutrición, Departamento de Salud Pública, Historia de la Ciencia y Ginecología, Universidad Miguel Hernández (UMH), 03550 Alicante, Spain; 3 CIBER Epidemiología y Salud Pública (CIBERESP), Instituto de Salud Carlos III, 28034 Madrid, Spain

**Keywords:** coffee, caffeinated, decaffeinated, mortality, cardiovascular disease, cancer

## Abstract

We assessed the association between usual coffee consumption and all-cause, cardiovascular (CV), and cancer mortality in an adult population in Spain, taking into account both the amount and type of coffee consumed. We used baseline data on coffee consumption and other personal variables, and the number of deaths during an 18-year follow-up period, for 1567 participants aged 20 years and older from the Valencia Nutrition Study in Spain. Total, caffeinated, and decaffeinated coffee consumption was assessed using a validated food frequency questionnaire. Cox regression models were used to estimate adjusted hazard ratios (HRs) and 95% confidence intervals (CIs). During the 18-year follow-up period, 317 died; 115 due to CV disease and 82 due to cancer. Compared with no-consumption, the consumption of ≤1 cup per day and >1 cup per day of coffee was associated with a lower risk of all-cause mortality, HR = 0.73 (95% CI: 0.56–0.97) and HR 0.56 (95% CI: 0.41–0.77), respectively. A lower cancer mortality was observed among drinkers of more than 1 cup per day compared with nondrinkers, HR 0.41 (95% CI 0.20–0.86). Regarding the type of coffee, only the overall consumption of caffeinated coffee was associated with lower all-cause mortality at 12 and 18 years of follow-up, HR = 0.66 (95% CI:0.46–0.94) and HR = 0.59 (95% CI: 0.44–0.79), respectively. In conclusion, this study suggests that the moderate consumption of coffee, particularly caffeinated coffee (range 1–6.5 cups per day), is associated with a lower all-cause and cancer mortality after a long follow-up period. No significant association was found between coffee consumption and CVD mortality.

## 1. Introduction

Coffee consumption is very prevalent worldwide and it has been associated with lower total mortality, although the association is not fully consistent and the evidence from Mediterranean countries is still scarce [1]. Coffee consumption has been associated with increased low-density lipoprotein (LDL-c) concentration [2], insulin resistance [3], increased blood pressure [4] and higher risk of some cardiovascular diseases (CVD) [4,5], although no long-term adverse effects have been consistently found with its consumption. Most of the recently published studies have reported inverse associations between habitual coffee consumption and the incidence of some diseases such as type 2 diabetes [6,7], mental illness [8,9], cardiovascular diseases [10,11,12], and cancer [10,13,14], all of which are major causes of mortality.

These findings are in agreement with the results obtained by Kim et al. [1] in a recent meta-analysis that included 40 studies and 3,852,651 subjects from different countries. In this meta-analysis, a non-linear association between coffee consumption and mortality from all causes, CVD, and cancer mortality was shown, with the lowest cancer mortality observed for the intake of two cups per day (RR = 0.96), the lowest CVD mortality for 2.5 cups per day (RR = 0.83), and the lowest all-cause mortality for 3.5 cups per day (RR = 0.85), with no additional reduction or increase in mortality with increasing coffee consumption [1].

The mechanisms by which coffee may reduce the risk of death are not well known, although it could be due to the antioxidant and anti-inflammatory effects of some of their components [10,11]. Coffee is rich in polyphenols, a group of compounds with antioxidant and anti-inflammatory activity which can be divided according to their chemical structure into flavonoids and non-flavonoids [15,16]. Some meta-analyses have shown that flavonoids and some non-flavonoids such as lignans with weak estrogen-like activity may have beneficial effects against cardiovascular disease and some cancers [15], although more evidence on specific compounds is still needed.

Evidence found in the few studies that have examined the role of coffee consumption on all-cause, CVD, and cancer mortality in Mediterranean populations with high life-expectancy and healthy diets is still insufficient. To the best of our knowledge, only two studies have specifically evaluated the association between coffee consumption and mortality in adults in Spain [17,18], and both have shown an inverse association between coffee consumption and total and CVD mortality [17,18]. In addition, in a recently published study with adults in Italy, a moderate consumption of 3–4 cups/day of coffee was associated with lower risk of all-cause and CVD mortality [19]. Thus, we assessed the association between coffee consumption and all-cause, CVD, and cancer mortality in a representative sample of an adult population in Valencia, Spain, taking into account both the amount and type of coffee.

## 2. Materials and Methods

### 2.1. Study Design and Population

Data for this study came from Valencia Nutrition Survey (VNS) conducted in 1994. Survey methods have been described in detail elsewhere [20]. Briefly, the VNS was a health, nutrition, and examination survey based on a representative sample, which enrolled 1811 adults in the Valencia Region aged 15 years and older (74.4% participation rate). Participants younger than 20 years and those with no information regarding coffee consumption were excluded from the present analysis. Thus, the final analysis was conducted with 1567 participants aged 20 years and above with complete information (718 men, 849 women). We obtained written informed consent from all participants, and the Ethical Committees of the Hospital of San Juan and the Miguel Hernandez University approved the study.

### 2.2. Coffee and Dietary Assessment

We collected the dietary information using a validated semi-quantitative food frequency questionnaire (FFQ). This FFQ was similar to the Willett questionnaire [21], which was adapted and validated in adult and elderly populations in Spain [22]. We used the FFQ in the VNS that had 93 food items and included nine sections for the main food groups: dairy, eggs, meat and fish, vegetables, fruits, breads and cereals, oils and fats, sweets and pastry, beverages, and processed foods. The validity and reproducibility of the FFQ has been described previously [22], which showed satisfactory reproducibility and validity. We compared the nutrients and food intake estimates in the adult population with those from four one-week dietary records. The average correlation coefficients for one-year validity and reproducibility of nutrient intakes were 0.47 and 0.40, respectively. We observed a good reproducibility for total coffee consumption, with a correlation coefficient of 0.60.

We asked subjects in our study how often, on average, they had consumed a standard portion size of each food item during the previous year. The FFQ had nine possible consumption frequencies, ranging from “never or less than once per month” to “six or more per day”. Two items were included to collect information about coffee consumption: one item for caffeinated coffee, and another item for decaffeinated coffee. We defined a cup of coffee using typical sizes (50 mL for espresso, or 125–150 mL for instant/brewed/ground coffee) and we calculated total coffee consumption in cups per day as the sum of decaffeinated and caffeinated coffee. We classified participants according to their total coffee consumption as non-drinkers, drinkers of ≤1 cup/day, and drinkers of >1 cup/day.

Adherence to a Mediterranean diet (MD) was estimated for each subject using the relative Mediterranean diet score (rMED) [23], which is a variation of the original Mediterranean diet score [24,25]. Instead of using the median to score each component, in the rMED, the intake in grams of each component (except for alcohol) is referred to per 1000 kcals/day and divided into tertiles. We assigned values of 0, 1, and 2 to the first, second, and third tertiles of intake, respectively, for the six components that form the MD. The six categories included fruits (including seeds and nuts), vegetables (excluding potatoes), fish, legumes, olive oil, and cereals (including whole grain). Dairy products and total meat (including processed meat) were negatively scored, probably because these components do not fit the MD (lower scoring for the higher intakes). Owing to the assumed beneficial effects of moderate alcohol consumption, we calculated it as a dichotomous variable: 2 points for moderate consumption (5–25 g/day for women and 10–50 g/day for men), and 0 points for higher or lower consumption. Finally, the rMED score was estimated for each participant by adding up the points of the 9 components. The scores ranged from 0–6 points (low adherence), 7–10 (medium adherence), to 11–18 points (high adherence). Nutrient values and energy intake were obtained from food composition tables from the U.S. Department of Agriculture [26] and other published Spanish sources [27].

### 2.3. Assessment of Mortality

We checked the information on the date and cause of deaths through the National Death Index from the Spanish Statistical Office and the Mortality Registry in the Valencia Region during the 18-year follow-up period.

We coded all causes of death according to version 10 of the International Classification of Diseases (ICD−10). Due to the small number of deaths, we grouped deaths in three major categories, which included cardiovascular disease (ICD−10: I00–I99), cancer (ICD−10 codes: C00–D49), and all-cause mortality. All-cause mortality category included deaths occurring from any cause as well as the first two categories.

### 2.4. Other Variables

Trained fieldworkers collected baseline information from all participants on socio-demographic and other lifestyles variables, including smoking habits, alcohol consumption, health status, physical activity, and chronic diseases, using structured questionnaires.

The following variables were considered in the analyses: sex (men, women), age (in years), educational level (<primary school; ≥primary school), body mass index (BMI) measured as weight in kilograms divided by the square of measured height in meters (<25 kg/m^2^, 25–30 kg/m^2^, ≥30 kg/m^2^), waist circumference (healthy range: 78–94 cm in men and 64–80 cm in women; moderate risk: 94–102 cm in men and 80–88 cm in women; and increased risk: >102 cm in men and >88 cm in women) [28], smoking status (never, ex-smoker, current), self-reported main physical activity at leisure-time (low, moderate –vigorous), total hours of TV watching per day, and total sleeping time in hours per day. We also collected the presence of pre-existing chronic disease at baseline, diabetes (no/yes), high blood cholesterol (no/yes), and high blood pressure(no/yes). In the adult population, previous studies have shown that a high level of agreement has been observed between self-reported diseases and those documented in the medical records [29,30].

### 2.5. Statistical Analysis

Statistical tests were bilateral, and signification was established at 0.05. We performed the analysis with the statistical software R.3.3.2 (R Foundation for Statistical Computing, Vienna, Austria, http://www.r-project.org, accessed on 1 April 2020)

We classified participants according to their total coffee consumption as non-drinkers, drinkers of up to 1 cup per day (range 0.1–1.0 cups), and drinkers of more than 1 cup per day (range 1.1–6.5). We also classified participants by the type of coffee in no coffee consumption, caffeinated, or decaffeinated consumption. Descriptive analysis of socio-demographic factors was performed between different coffee consumption using percentages and Chi-squared tests to describe and compare categorical variables, and for continuous variables, we used means, standard deviations, and ANOVA tests.

We estimated person-years for each participant of follow-up from the date of the interview at baseline to the date of death or completion of the 6-, 12- and 18-year follow-up, whichever came first. We analyzed the association and risk of mortality at 6, 12 and 18 years of follow-up (ad hoc division) and total, caffeinated, and decaffeinated coffee consumption to explore the potential short, medium, and long-term effects of coffee, adjusting for other variables. We obtained hazard ratios (HRs) and 95% confidence intervals (95%CI) by Cox’s proportional hazard for each category of coffee consumption in comparison to the lower category (no consumption, ≤1 cup/day, >1 cup/day) from all causes of mortality, CVD, and cancer mortality.

Two models were presented, one adjusted for age and sex, and multivariable analyses were performed, in which we further adjusted for several factors considered as potential confounders established in the literature and those variables showing *p*-values < 0.20 in bivariate analysis. We adjusted by: sex, age (in years), education level (<primary school; ≥primary school), BMI (<25 kg/m^2^, 25–30 kg/m^2^, ≥30 kg/m^2^), waist circumference (healthy range: 78–94 cm in men and 64–80 cm in women; moderate risk: 94–102 cm in men and 80–88 cm in women; and increased risk: >102 cm in men and >88 cm in women) [28], smoking (never, ex-smoker, current), self-reported main physical activity at leisure-time (very low, mostly sitting position; low, moderate–vigorous), adherence to a Mediterranean diet (rMED), hours of TV watching per day, total sleeping time in hours per day, and diabetes (no/yes), high blood cholesterol (no/yes), and high blood pressure (no/yes).

The non-zero slope of the scaled Schoenfeld residuals on the time function suggested that the proportional hazard assumption was met. We calculated the likelihood ratio test (LRT) to estimate the overall significance of coffee consumption as a categorical variable, and we calculated trend tests to evaluate the dose–response for total coffee consumption as a continuous term.

## 3. Results

The main characteristics of the study population according to coffee consumption are shown in Table 1. Of 1567 participants, 78% were coffee drinkers, of whom 37.7% were drinkers of up to 1 cup per day, and 40.3% reported drinking more than one cup of coffee per day. In general, participants who consumed >1 cup/daily were more likely to be current smokers, have a higher education, spend less time watching television, and have a lower prevalence of self-reported diabetes and hypertension.

**Figure 1:**
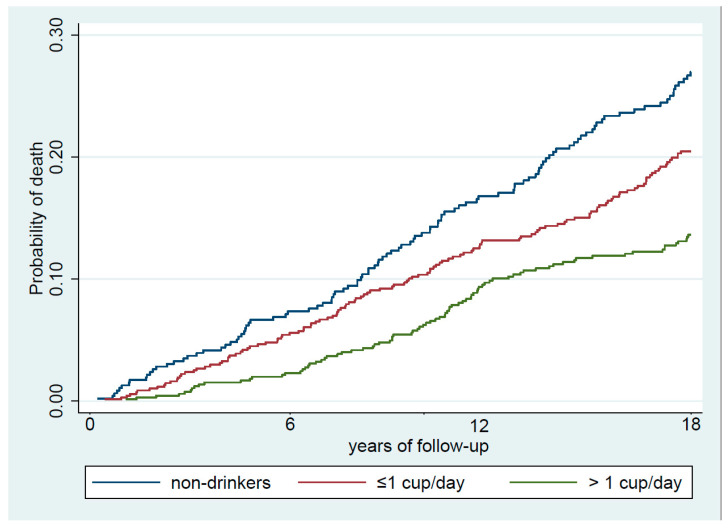
Cumulative incidence curves of death after 18 years of follow-up, according to total coffee consumption for all-cause mortality in participants from the Valencia Nutritional Survey in Spain (*n* = 1567).

As shown in Table 2, during the first six years of follow-up (9169.4 person-years), 85 deaths occurred. Of these deaths, 31 (36.4%) were from CV diseases and 25 (29.4%) were due to cancer. In the 12 years of follow-up (17,693.7 person-years), we documented 216 deaths; 77 (35.6%) due to CV diseases, and 56 (25.9%) to cancer. Finally, during the total 18 years of follow-up (25,526.9 person-years), we documented 317 deaths; 115 (36.3%) due to CVD, and 82 (25.9%) to cancer. Curves of cumulative incidence for all-cause mortality during the study period according to coffee consumption are shown in Figure 1. Coffee drinkers showed lower incidence of mortality than nondrinkers in cumulative incidence curves.

Table 2 shows the HRs of all-cause, CVD, and cancer mortality for coffee consumption. Coffee consumption was inversely associated with all-cause mortality during the follow-up period. After six years of follow-up, compared with non-drinkers, drinkers of ≤1 cup of regular coffee had a 22% non-significant lower risk of death, and those who consumed more than one cup/day showed a 56% reduced risk of death, HR = 0.44 (95% CI: 0.22–0.85). Similarly, at 12 years of follow-up, lower all-cause mortality was observed among drinkers of more than one cup per day, HR = 0.67 (95% CI: 0.46–0.98). After 18 years of follow-up, drinkers of up to one cup per day and those of more than one cup per day showed a lower risk of all-cause mortality compared with non-drinkers, HR = 0.73 (95% CI: 0.56–0.97) and HR = 0.56 (95% CI: 0.41–0.77), respectively, with a significant dose–response trend (*p*-trend = 0.001). The number of deaths by cancer and CV diseases after 6 and 12 years of follow-up was too low, although after 18 years of follow-up, an inverse association was observed for cancer mortality among those who consumed more than one cup per day, HR 0.41 (95% CI: 0.20–0.86).

The association between type of coffee consumption and mortality at 6, 12 and 18 years of follow-up is shown in Table 3. Compared with non-drinkers, drinkers of caffeinated coffee at 12 and 18 years of follow-up showed lower risks of all-cause mortality; HR = 0.66 (95% CI: 0.46–0.94) and HR = 0.59 (95% CI: 0.44–0.79), respectively. There was some evidence for an inverse association between caffeinated coffee and cancer mortality at 18 years of follow-up (*p* = 0.10). No statistically significant association was observed between decaffeinated coffee consumption and all-cause, CVD, or cancer mortality during the study period.

## 4. Discussion

In this study, we have shown an inverse association between baseline coffee consumption and all-cause and cancer mortality in an adult Mediterranean population after 18 years of follow-up. Compared with no-consumption, coffee consumption of one or fewer cups per day was associated with a 27% reduction in all-cause mortality, and the consumption of more than one cup/day (range 2–6.5 cups/day) was associated with a 44% reduction in all-cause mortality. The consumption of more than one cup of coffee per day was also associated with a 59% reduction in cancer mortality after 18 years of follow-up. We did not observe this protective effect for CVD mortality. Regarding the type of coffee, the protective effect was observed only between caffeinated coffee and all causes of mortality after 12 and 18 years of follow-up.

The inverse association between coffee consumption and all-cause mortality observed in our study is consistent with the results from previous meta-analyses in adult populations [11,31,32,33,34], and also with results observed in subsequent prospective studies carried out in the United States [12,35], Europe [36,37], and Asia [38]. Nevertheless, this association has scarcely been evaluated in adult populations from Mediterranean countries, where the high adherence to a Mediterranean dietary pattern may reduce mortality from all causes [39]. Although our results may not seem fully innovative with respect to other studies that have previously reported a protective effect of coffee consumption, the interest and the novelty of this study may still be sustained by the fact that it is the first study to evaluate the association between coffee consumption and all-cause, CVD, and cancer mortality in adults aged 20 years and older of a Mediterranean country, i.e., Spain. As far as we know, only three studies have specifically explored the association between coffee consumption and total mortality with Mediterranean populations. In one previous study carried out by our group with an elderly population in Valencia, an inverse association was observed with CVD but not for all causes of mortality [17]. In the SUN study, a prospective cohort study with university graduate participants, an inverse association was also observed for all-cause mortality among participants who consumed ≥4 cups/day of coffee [18]. Finally, a recently published prospective cohort study with Italian adults reported that moderate consumption of 3–4 cups/day of Italian-style coffee was associated with lower risk of all-cause and, specifically, of CVD mortality [19].

Most studies examining the association between coffee consumption and CV diseases have reported inverse associations [12,17,36,37], although in some studies the association was non-statistically significant [31,40]. In this study, we have found evidence of an inverse association, which is also consistent with a study we carried out in elderly population [17]. Regarding cancer mortality, the inverse association we found is consistent with previous studies in different populations [35,37,38], although there are some studies showing no association [12,36]. Other studies have shown an inverse association with specific types of cancer [41,42]; however, two recently published meta-analyses provided evidence for an inverse association with cancer mortality [1,11]. Overall, the present evidence suggests that moderate coffee consumption may reduce cancer mortality, as shown in our study that the consumption of more than one cup per day was associated with a 59% decreased risk of cancer mortality.

When we explored the association by type of coffee, we found an inverse association between caffeinated coffee and all-cause mortality at 12 and 18 years of follow-up. Although some studies show that caffeine contained in coffee may produce some adverse effects in the central nervous system and cardiovascular system [4,5,43], several cohort studies have found that moderate caffeine consumption is associated with a decreased risk of all-cause mortality in adult populations [12,35]. Most of these studies have also reported an inverse association with decaffeinated coffee consumption, which we did not observe in our study, although our study had the lack of power to detect an association because the number of events by categories of decaffeinated coffee was small, and decaffeinated coffee was also less frequently consumed than caffeinated coffee.

Several biological mechanisms have been proposed to explain why coffee may decrease the risk of mortality. Coffee is a rich source of antioxidant components such as caffeine, chlorogenic acid, melanoidins, cafestol, kahweol, and trigonelline, as well as other polyphenol compounds that may have important beneficial effects on inflammation, and beneficial effects have been shown against total mortality, cardiovascular disease and some cancers [15]. Firstly, caffeine and chlorogenic acid contents in coffee produce an inhibition of peroxidation of LDL-c which prevents the development of atherosclerosis and decreases oxidative stress, preventing endothelial dysfunction [44,45]. Moreover, other phenolic compounds and substances such as trigonelline or magnesium may improve insulin sensitivity and glucose resistance [7]. Finally, coffee may produce biological anticarcinogenic effects, including inhibition of the enzyme responsible for carcinogen activation, the stimulation of intracellular antioxidant defense mechanisms, and inhibition of DNA methylation that manages the inactivation of tumorigenic process and apoptosis [45]. Thus, compounds of coffee might play a beneficial role in health, mediating not only the association between long-term coffee consumption and risk of all-cause mortality, but also with cancer mortality.

The current study has several limitations. Firstly, we were not able to control for possible changes in coffee consumption during the follow-up; however, coffee consumption is a habit adopted in adult life that rarely changes over time, and the self-reported consumption could be a valid method to assess usual long-term coffee consumption [12,46]. Secondly, it is possible that pre-existing chronic illness at baseline might cause higher mortality and be also associated with lower or non-coffee consumption. When we repeated the analyses excluding deaths in the first and second year of the follow-up and adjusted for the self-reported pre-existing chronic diseases at baseline, the associations remained basically unchanged (data not shown). Thirdly, although participants were volunteers in a nutrition survey and some response bias may be possible, it is unlikely that coffee consumption was influential in the participation rate in the study, and coffee consumption among our participants was similar to that found in other Spanish studies [17,18]. Additionally, we did not collect information on the method of coffee preparation, but a previous study showed that unfiltered coffee is the most widely consumed in Spain [47]. Lastly, a point to consider is the small sample size, which may have limited the statistical power to detect some associations as significant (e.g., CV diseases); however, the follow-up period was long enough to detect significant associations with all-cause and cancer mortality.

Our study has several strengths, however. We used a well-defined population comprising participants aged 20 or above from a well-defined Mediterranean area from which high-quality information was collected at baseline by trained fieldworkers using standardized protocols and validated questionnaires. In addition, the information on coffee consumption was collected before the outcome occurred; thus, any misclassification in coffee consumption categories, if any, should be non-differential, and could therefore lead to an underestimation of the effects of coffee on mortality.

## 5. Conclusions

In conclusion, this study suggests that the moderate consumption of coffee, particularly caffeinated coffee (range 1–6.5 cups per day), is associated with a lower all-cause and cancer mortality after a long follow-up period. These findings are consistent with previous studies, although they add new evidence from a Mediterranean adult population. Thus, coffee consumption could be promoted as part of a healthy Mediterranean lifestyle, although further long-term longitudinal studies collecting information on the amount and type of coffee should add valuable information regarding its beneficial effects.

## Figures and Tables

**Table 1:** Socio-demographic and lifestyle characteristics according to total coffee consumption among participants aged 20 years and above of the EUREYE-Spain and the Valencia Nutrition Study in Spain (*n* = 1567).

	Coffee Consumption				
	Total	No.	≤1 Cup/Day	>1 Cup/Day	*p* ^1^
Study, *n* (%)	1567 (100.0)	345 (22.0)	591 (37.7)	631 (40.3)	<0.001
Sex, *n* (%)					
Men	718 (45.8)	147 (42.6)	274 (46.4)	297 (47.1)	
Women	849 (54.2)	198 (57.4)	317 (53.6)	334 (52.9)	0.39
Age, mean (SD)	45.9 (18.0)	48.6 (20.6)	46.0 (18.8)	44.4 (15.5)	0.002
Education Level, *n* (%)					
<Primary school	714 (45.6)	177 (51.3)	290 (49.1)	247 (39.1)	
≥Primary school	853 (54.4)	168 (48.7)	301 (50.9)	384 (60.9)	<0.001
Body Mass Index kg/m^2^, *n* (%)					
<25 kg/m^2^	650 (41.7)	139 (40.4)	254 (43.3)	257 (40.9)	
25–30 kg/m^2^	623 (40.0)	126 (36.6)	232 (39.5)	265 (42.2)	
≥30 kg/m^2^	286 (18.3)	79 (23.0)	101 (17.2)	106 (16.9)	0.11
Waist circumference *, *n* (%)					
Healthy range	582 (37.6)	122 (36.0)	222 (37.9)	238 (38.2)	
Moderate risk	373 (24.1)	68 (20.1)	137 (23.4)	168 (27.0)	
Increased risk	592 (38.3)	149 (44.0)	226 (38.6)	217 (34.8)	0.05
Smoking Status, *n* (%)					
Never	775 (49.5)	223 (64.6)	308 (52.1)	244 (38.7)	
Ex-smoker	262 (16.7)	44 (12.8)	101 (17.1)	117 (18.5)	
Current	550 (33.8)	78 (22.6)	182 (30.8)	270 (42.8)	<0.001
Diabetes ^3^ (yes), *n* (%)	121 (7.7)	41 (11.9)	43 (7.3)	37 (5.9)	0.003
Cholesterol ^3^ (yes), *n* (%)	207 (13.2)	50 (14.5)	65 (11.0)	92 (14.6)	0.13
Hypertension ^3^ (yes), *n* (%)	280 (17.9)	92 (26.7)	100 (16.9)	88 (13.9)	<0.001
Physical activity at leisure time, *n* (%)					
Low	609 (39.6)	139 (41.4)	218 (37.4)	252 (40.6)	
Moderate–vigorous	930 (60.4)	197 (58.6)	365 (62.6)	368 (59.4)	0.38
TV watching, hours/day, mean (SD)	2.5 (1.77)	2.7 (2.0)	2.5 (1.6)	2.3 (1.7)	0.03
Sleeping time, hours/day, mean (SD)	7.5 (1.4)	7.5 (1.6)	7.5 (1.4)	7.4 (1.3)	0.57
rMED, mean (SD)	8.2 (2.6)	8.3 (2.6)	8.3 (2.6)	8.1 (2.6)	0.41

Abbreviations: VNS, Valencia Nutrition Survey; SD, standard deviation; BMI, body mass index; rMED, relative Mediterranean dietary index. ^1^ *p*-value (*p*) from chi-squared test (categorical variables) and ANOVA (continuous variables). ^*^ Waist circumference: healthy range (78–94 cm in men and 64–80 cm in women), moderate risk (94–102 cm in men and 80–88 cm in women), increased risk (>102 cm in men and >88 cm in women). ^3^ Self-reported diabetes (no/yes), high cholesterol (no/yes) and hypertension (no/yes).

**Table 2:** Associations between the level of total coffee consumption and all-cause, cardiovascular disease, and cancer mortality among participants of the Valencia Nutrition Survey in Spain.

Coffee Consumption					
	No.	≤1 Cup/Day	>1 Cup/Day	*p*-Value ^2^	*p*-Trend ^3^
	**Follow-up at 6 Years**				
All-cause mortality (*n*, %)	345 (22.0)	591 (37.7)	631 (40.3)		
Deaths, *n*	33	37	15		
Person-years	1971.3	3450.5	3747.6		
HR (95% CI)					
Age and sex adjusted	1.00	0.72 (0.45–1.15)	0.38 (0.20–0.72)		
Multivariable ^1^	1.00	0.78 (0.46–1.30)	0.44 (0.22–0.85)	0.01	0.04
CVD (*n*, %)	325 (21.5)	566 (37.4)	622 (41.1)		
Deaths, n	13	12	6		
Person-years	1918.8	3369.9	3713.5		
HR (95% CI)					
Age and sex adjusted	1.00	0.66 (0.30–1.46)	0.44 (0.17–1.20)		
Multivariable ^1^	1.00	0.76 (0.32–1.83)	0.42 (0.14–1.26)	0.11	0.27
Cancer (*n*, %)	319	569	619		
Deaths, *n*	7	15	3		
Person-years	1891.8	3371.8	3711.0		
HR (95% CI)					
Age and sex adjusted	1.00	1.36 (0.54–3.35)	0.34 (0.09–1.34)		
Multivariable ^1^	1.00	1.54 (0.58–4.08)	0.45 (0.10–1.90)	0.35	0.10
	**Follow-up at 12 Years**				
All-cause mortality (*n*, %)	345 (22.0)	591 (37.7)	631 (40.3)		
Deaths, *n*	70	84	62		
Person-years	3734.7	6633.5	7325.5		
HR (95% CI)					
Age and sex adjusted	1.00	0.76 (0.55–1.05)	0.70 (0.49–1.00)		
Multivariable ^1^	1.00	0.75 (0.53–1.06)	0.67 (0.46–0.98)	0.04	0.10
CVD (*n*, %)	302 (21.1)	530 (37.1)	596 (41.7)		
Deaths, *n*	27	23	27		
Person-years	3475.2	6229.7	7048.4		
HR (95% CI)					
Age and sex adjusted	1.00	0.62 (0.35–1.09)	1.03 (0.59–1.79)		
Multivariable ^1^	1.00	0.63 (0.34–1.19)	1.00 (0.54–1.89)	0.99	0.23
Cancer (*n*, %)	289 (20.5)	537 (38.2)	581 (41.3)		
Deaths, *n*	14	30	12		
Person-years	3385.2	6268.7	6927.5		
HR (95% CI)					
Age and sex adjusted	1.00	1.34 (0.70–2.55)	0.70 (0.32–1.54)		
Multivariable ^1^	1.00	1.37 (0.69–2.72)	0.51 (0.20–1.27)	0.16	0.03
	**Follow-up at 18 Years**				
All-cause mortality (*n*, %)	345 (22.0)	458 (37.7)	631 (40.3)		
Deaths, *n*	107	126	84		
Person-years	5273.3	9575.5	10,678.1		
HR (95% CI)					
Age and sex adjusted	1.00	0.72 (0.56–0.94)	0.58 (0.43–0.79)		
Multivariable ^1^	1.00	0.73 (0.56–0.97)	0.56 (0.41–0.77)	<0.001	0.001
CVD (*n*, %)	276 (20.2)	504 (36.9)	585 (42.9)		
Deaths, *n*	38	39	38		
Person-years	4621.5	8764.2	10,242.5		
HR (95% CI)					
Age and sex adjusted	1.00	0.61 (0.38–0.96)	0.80 (0.49–1.27)		
Multivariable ^1^	1.00	0.66 (0.40–1.07)	0.71 (0.41–1.20)	0.19	0.22
Cancer (*n*, %)	259 (19.4)	508 (38.1)	565 (42.4)		
Deaths, *n*	21	43	18		
Person-years	4472.8	8759.7	10,027.4		
HR (95% CI)					
Age and sex adjusted	1.00	0.93 (0.55–1.58)	0.47 (0.25–0.90)		
Multivariable ^1^	1.00	1.01 (0.57–1.79)	0.41 (0.20–0.86)	0.01	0.01

^1^ Cox regression model adjusted for age, sex, educational level (<Primary, ≥Primary), BMI (<25, 25.0–29.9, ≥30), waist circumference (healthy, moderate and increased risk), sleeping time (h/day), smoking status (current; past and never), self-reported diabetes (no/yes), high cholesterol (no/yes), hypertension (no/yes), relative Mediterranean diet, physical activity at leisure time (low, moderate–high) and TV watching (h/day). ^2^ *p*-value from the likelihood ratio test. ^3^ *p*-value from the *p*-trend test.

**Table 3:** Associations between type of coffee consumption and all-cause, cardiovascular disease, and cancer mortality among participants of the Valencia Nutrition Survey in Spain.

Coffee Consumption				
	No.	Decaffeinated Coffee ^2^ (Range 0.1–6.5 Cups/Day)	Caffeinated Coffee ^2^ (Range 0.1–6.5 Cups/Day)	*p*-Value ^3^
	**Follow-up at 6 years**			
All-cause mortality (*n*, %)	345 (22.0)	308 (19.7)	914 (58.3)	
Deaths, *n*	33	24	28	
Person-years	1971.3	1783.4	5414.7	
HR (95% CI)				
Age and sex adjusted	1.00	0.61 (0.36–1.04)	0.55 (0.33–0.94)	
Multivariable ^1^	1.00	0.66 (0.37–1.18)	0.62 (0.35–1.10)	0.20
CVD (*n*, %)	325 (21.5)	295 (19.5)	893 (59.0)	
Deaths, *n*	13	11	7	
Person-years	1918.8	1741.8	5341.6	
HR (95% CI)				
Age and sex adjusted	1.00	0.72 (0.32–1.63)	0.42 (0.16–1.08)	
Multivariable ^1^	1.00	0.66 (0.26–1.65)	0.55 (0.20–1.52)	0.46
Cancer (*n,* %)	319 (21.2)	293 (19.4)	895 (59.4)	
Deaths, *n*	7	9	9	
Person-years	1891.8	1736.6	5346.1	
HR (95% CI)				
Age and sex adjusted	1.00	1.03 (0.38–2.80)	0.81 (0.38–2.81)	
Multivariable ^1^	1.00	1.42 (0.50–4.09)	0.89 (0.29–2.69)	0.65
	**Follow-up at 12 years**			
All-cause mortality (*n*, %)	345 (22.0)	308 (19.7)	914 (58.3)	
Deaths, *n*	70	65	81	
Person-years	3734.7	3361.1	10,598.0	
HR (95% CI)				
Age and sex adjusted	1.00	0.81 (0.58–1.14)	0.68 (0.49–0.95)	
Multivariable ^1^	1.00	0.80 (0.55–1.15)	0.66 (0.46–0.94)	0.08
CVD (*n*, %)	302 (21.1)	271 (19.0)	855 (59.9)	
Deaths, *n*	27	28	22	
Person-years	3475.2	3112.1	10,165.9	
HR (95% CI)				
Age and sex adjusted	1.00	0.92 (0.54–1.56)	0.64 (0.35–1.15)	
Multivariable ^1^	1.00	0.83 (0.45–1.51)	0.71 (0.35–1.41)	0.60
Cancer (*n*, %)	289 (20.5)	261 (18.6)	857 (60.9)	
Deaths, *n*	14	18	24	
Person-years	3385.2	3029.4	10,166.8	
HR (95% CI)				
Age and sex adjusted	1.00	1.16 (0.58–2.35)	1.01 (0.51–2.00)	
Multivariable ^1^	1.00	1.24 (0.58–2.69)	0.88 (0.41–1.87)	0.65
	**Follow-up at 18 years**			
All-cause mortality (*n*, %)	345 (22.0)	308 (19.7)	914 (58.3)	
Deaths, *n*	107	95	115	
Person-years	5273.3	4746.4	15,507.1	
HR (95% CI)				
Age and sex adjusted	1.00	0.77 (0.58–1.01)	0.59 (0.45–0.77)	
Multivariable ^1^	1.00	0.76 (0.56–1.03)	0.59 (0.44–0.79)	0.002
CVD (*n*, %)	276 (20.2)	255 (18.7)	834 (61.1)	
Deaths, *n*	38	42	35	
Person-years	4621.5	4255.6	14,751.1	
HR (95% CI)				
Age and sex adjusted	1.00	0.80 (0.51–1.24)	0.57 (0.35–0.92)	
Multivariable ^1^	1.00	0.69 (0.42–1.14)	0.70 (0.38–1.14)	0.23
Cancer (*n*, %)	259 (19.4)	242 (18.2)	831 (62.4)	
Deaths, *n*	21	29	32	
Person-years	4472.8	4113.1	14,674.085	
HR (95% CI)				
Age and sex adjusted	1.00	0.95 (0.54–1.69)	0.59 (0.33–1.03)	
Multivariable ^1^	1.00	1.01 (0.54–1.87)	0.57 (0.31–1.08)	0.11

Abbreviations: HR, hazard ratio; CI, confidence interval; CVD, cardiovascular disease. ^1^ Cox regression model adjusted for age, sex, educational level (<Primary, ≥Primary), BMI (<25, 25.0–29.9, ≥30), waist circumference (healthy range, moderate risk and increased risk), sleeping time (h/day), smoking status (current; past and never), self-reported diabetes (no/yes), high cholesterol (no/yes), hypertension (no/yes), relative Mediterranean diet, physical activity at leisure time (low, moderate–high) and TV watching (h/day). ^2^ Any coffee consumption. ^3^ *p*-value from the likelihood ratio test.

## Data Availability

The data presented in this study are available on request from the corresponding author. The data are not publicly available due to confidentiality and ethical reasons.
